# Effect of Semaglutide on Subclinical Atherosclerosis and Cardiometabolic Compensation: A Real-World Study in Patients with Type 2 Diabetes

**DOI:** 10.3390/biomedicines11051362

**Published:** 2023-05-04

**Authors:** Angelo Maria Patti, Rosaria Vincenza Giglio, Alberto Allotta, Andreina Bruno, Tommaso Di Bella, Anca Pantea Stoian, Marcello Ciaccio, Manfredi Rizzo

**Affiliations:** 1 Internal Medicine Unit, “Vittorio Emanuele II” Hospital, 91022 Castelvetrano, Italy; albertoallotta@libero.it; 2 Clinical Molecular Medicine and Laboratory Medicine, Department of Biomedicine, Neurosciences and Advanced Diagnostics, Institute of Clinical Biochemistry, University of Palermo, 90127 Palermo, Italy; giglio.rosaria.vincenza@gmail.com (R.V.G.); marcello.ciaccio@unipa.it (M.C.); 3 Department of Laboratory Medicine, University Hospital “P. Giaccone”, 90127 Palermo, Italy; 4 Institute of Translational Pharmacology (IFT), National Research Council (CNR), 90146 Palermo, Italy; andreina.bruno@ift.cnr.it; 5 Geriatrics Complex Operative Units, Vittorio Emanuele III Hospital, 91018 Salemi, Italy; tommaso.dibella@asptrapani.it; 6 Department of Diabetes, Nutrition and Metabolic Diseases, “Carol Davila” University of Medicine, 050474 Bucharest, Romania; ancastoian@yahoo.com (A.P.S.); manfredi.rizzo@unipa.it (M.R.); 7 Department of Health Promotion, Mother and Child Care, Internal Medicine and Medical Specialties (ProMISE) “G. D’Alessandro”, University of Palermo, 90127 Palermo, Italy

**Keywords:** semaglutide, carotid intima-media thickness, cardiovascular risk, type 2 diabetes, small dense LDL, nonalcoholic fatty liver disease

## Abstract

Background: Semaglutide is a recently approved glucagon-like peptide-1 receptor agonist. Several trials reported the protective effect of injectable semaglutide on cardiovascular (CV) risk by reducing major adverse cardiovascular events in type 2 diabetes patients. Strong preclinical evidence supports the CV benefits of semaglutide through an effect on atherosclerosis. However, scant evidence is available about the protective mechanisms of semaglutide in clinical practice. Methods: A retrospective observational study was conducted among consecutive type 2 diabetes patients treated with injectable semaglutide in Italy between November 2019 and January 2021 when the drug was first available in the country. The primary aims were the assessment of the carotid intima-media thickness (cIMT) and hemoglobin A1c (HbA1c) levels. The secondary aims were the evaluation of anthropometric, glycemic, and hepatic parameters and plasma lipids, including the assessment of the triglyceride/high-density lipoprotein ratio as an indirect marker of atherogenic small, dense low-density lipoprotein particles. Results: Injectable semaglutide reduced HbA1c and cIMT. An improvement in CV risk factors and the triglyceride/high-density lipoprotein ratio was reported. Moreover, through correlation analyses, we found that hepatic fibrosis and steatosis indices and the anthropometric, hepatic, and glycemic parameters, as well as plasma lipids, were unrelated to the variations in cIMT and HbA1c. Conclusions: Our findings suggest the effect of injectable semaglutide on atherosclerosis as a key CV protective mechanism. Considering the favorable effects on atherogenic lipoproteins and hepatic steatosis indices, our results support the pleiotropic effect of semaglutide beyond glycemic control.

## 1. Introduction

Type 2 diabetes (T2D) is a metabolic condition associated with an increased cardiovascular (CV) risk [1]. Innovative diabetes therapies, such as glucagon-like peptide-1 receptor agonists (GLP-1RA), have shown additional glycemic preventive effects beyond the nonglycemic activity, acting on several cardio-metabolic parameters and restoring the balance of several interlinking aspects of metabolism [2]. These activities have been associated with several molecular mechanisms, mainly related to the action on the exposure of the LDL receptor (LDL-R), endothelial dysfunction, activation of macrophages, lipid oxidation, suppression of macrophage foam cells, and deposition of extracellular lipids [3,4,5]. Consequently, GLP-1RA therapies can contribute to reducing and/or preventing the incidence of CV events in daily clinical practice and slowing the development of vascular injuries, the main source of early morbidity and mortality in diabetic subjects [6].

Semaglutide (Novo Nordisk, Bagsværd, Denmark) is the most recently approved agent among GLP-1RAs, developed to treat patients with T2D who are not achieving their hemoglobin A1c (HbA1c) goals with other anti-hyperglycemic medications [7]. Semaglutide is a long-acting GLP-1RA with a 1-weekly extended release; it has been on the market in Europe since 2018 and is the only GLP-1RA currently available as both a subcutaneous and oral formulation [8,9].

The Semaglutide Unabated Sustainability in Treatment of Type 2 Diabetes (SUSTAIN) program, comprising 10 clinical trials that compared semaglutide to a placebo or other antidiabetic treatments, provided evidence for the effectiveness of injectable semaglutide as a monotherapy or an add-on therapy [10]. A reduction in HbA1c up to 1.5–1.8%, a reduction in major adverse cardiovascular (MACE) events rates, kidney prevention, and weight loss after the semaglutide treatment were reported [11,12,13,14,15], as well as an overall favorable risk/benefit profile [7].

In particular, in the SUSTAIN-6 trial, the protective effect on the CV risk of injectable semaglutide is widely investigated, and a significant reduction in MACE is reported in the treated patients versus placebo [10,16]. Strong preclinical evidence supports the CV benefits of semaglutide through an effect on atherosclerosis. However, scant evidence is available about the protective mechanisms of semaglutide in clinical practice [17,18].

This real-world observational study aims to expand the knowledge about the injectable semaglutide treatment in clinical practice and to explore its effect on carotid intima-media thickness (IMT), a surrogate marker of subclinical atherosclerosis, and on cardiometabolic risk factors in patients with T2D.

## 2. Patients and Methods

### 2.1. Study Design

This was a monocenter, retrospective observational study conducted at the diabetes outpatient of “Vittorio Emanuele II” Hospital of Castelvetrano (Trapani, Sicily, Italy). The study involved consecutive patients with an established diagnosis of T2D treated with injectable semaglutide (Ozempic^®^; Novo Nordisk; Denmark) between November 2019 and January 2021.

This study was notified to the Ethics Committee of the Provincial Sanitary Agency (ASP) of Trapani and performed in accordance with the current legislation on observational studies and the Declarations of Helsinki. Patients gave their consent to the use of medical records for research purposes. For patients who did not sign the informed consent because they did not perform any visit after the notification of this retrospective study, the data processing was carried out after a formal request to the ethics committee.

### 2.2. Patients

T2D patients aged > 18 years on a weekly treatment with subcutaneous semaglutide were considered. According to the clinical practice, subcutaneous semaglutide was administered with a starting dose of 0.25 mg/week for the first month and then 0.50 mg/week for the next 3 months. During this study, any dosage from concomitant therapies remained unchanged in order to avoid potential confounders. All subjects were naïve to GLP-1RAs therapy. Exclusion criteria included excessive alcohol use, pregnancy, severe hepatic or kidney disease, serious infections, and malignancies.

### 2.3. Study Measures

The following data were collected at the baseline: clinical history, drug history, anthropometric parameters (weight, waist, and BMI), biochemical parameters (HbA1c, glycemia, plasma lipids, liver function parameters), and arterial wall thickness in the carotid arteries (cIMT). Blood samples were taken after a 12 h overnight fast. Biochemical parameters were measured by standard enzymatic colorimetric methods, while LDL-cholesterol was calculated using the Friedewald formula. The cIMT was assessed with real-time B-mode ultrasound analysis according to routine clinical practice. Examinations were performed by a single examinator using a single sonographer (Philips HDI 5000; Philips, Italy; with a probe of 7.5–10.0 MHz) in a blinded manner. As previously reported, the ultrasound examination was standardized with fixed insonation angles [19].

This study’s primary aims were to assess cIMT and HbA1c levels. The secondary purposes were evaluating the anthropometric parameters, glycemic parameters, plasma lipids and hepatic parameters, and the triglycerides (TG)/HDL ratio. Measures were collected after 4 months of treatment with semaglutide and compared with baseline values. Analyses were conducted both in the overall population and stratifying patients in three groups according to the disease duration: early diabetes stage (<10 years), medium diabetes stage (up to 15 years), and late diabetes stage (>15 years).

Variations in the hepatic steatosis markers (including fatty liver index [FLI] and hepatic steatosis index [HSI]), hepatic fibrosis markers (including AST/ALT ratio and BARD—body mass index, AST/ALT ratio, T2D—score), and triglyceride glucose (TyG) index were calculated in the overall population in the study period [20,21].

The correlation between these markers and changes in cIMT and HbA1c was assessed, as well as the correlation between anthropometric, glycemic, and hepatic parameters and plasma lipids and changes in cIMT and HbA1c. Variations in insulin dose during the observational period were also evaluated.

The observation phase lasted 4 months, as this was the first prescription of semaglutide (subject to a therapeutic plan) in Sicily at the moment of this study [22].

### 2.4. Statistical Analysis

Continuous variables were shown as mean and standard deviation and categorical variables as number and percentage. McNemar’s test was used to compare categorical variables within groups. The paired samples *t*-test was used to compare continuous variables within groups. The ANOVA test was used to compare continuous variables among groups. Univariate analysis of the variance was used to analyze the correlation between the primary endpoints and glycemic, hepatic, and plasma lipids parameters with the primary endpoints as the dependent variable and the parameters as covariates.

For the calculation of the triglyceride-glucose index (TyG), the following formula was used: ln [triglyceride (mg/dL) × fasting glucose (mg/dL)/2]. Values smaller than 4.5 are considered normal.

The following formula was used to calculate the hepatic steatosis index (HSI): 8 × AST/ALT + BMI (+2 if type 2 diabetes, yes, +2 if female). Values smaller than 30 indicate an absence of steatosis and values larger than 36 indicate steatosis.

The AST/ALT ratio was calculated; a value smaller than 1 indicates liver damage.

The following formula was used to calculate the BARD index: AST/ALT ratio ≥ 0.8 → 2 points, BMI ≥ 28 → 1 point; diabetes → 1 point. Values smaller than 2 are considered negative predictors of advanced fibrosis.

For the calculation of the fatty liver index (FLI), the following formula was used:

$$\frac{\left(e^{0.953}\times \log_{e}\left(triglycerides\right)+0.139\times BMI+0.718\times \log_{e}\left(GGT\right)+0.053\times waist\;circ-15.745\right)}{1+\left(e^{0.953}\times \log_{e}\left(triglycerides\right)+0.139\;\times BMI\;+\;0.718\;\times \log_{e}\left(GGT\right)+0.53\times waist\;circ-15.745\right)}\times 100$$

Values smaller than 30 indicate the absence of steatosis, and values larger than 60 indicate the presence of steatosis.

## 3. Results

### 3.1. Study Population and Clinical Characteristics

A total of 40 patients were included in this study. Baseline demographics, clinical characteristics, and concomitant therapies to semaglutide are presented in Table 1. Thirty-two percent of subjects were habitual smokers, and eighty-five percent suffered from hypertension. In total, 85% of patients were on metformin therapy (doses ranging from 500 to 3000 mg daily). A total of 12% of patients were on insulin therapy (dosage ranging from 12 to 40 IU). A total of 86% of patients were already taking anti-hypertensive or lipid-lowering drugs. About half of the patients (47%) were in the late diabetes stage. Baseline demographics and clinical characteristics of patients stratified according to the disease duration are provided in Supplementary Table S1.

### 3.2. Effectiveness Parameters

#### 3.2.1. Carotid IMT

**Figure 1:**
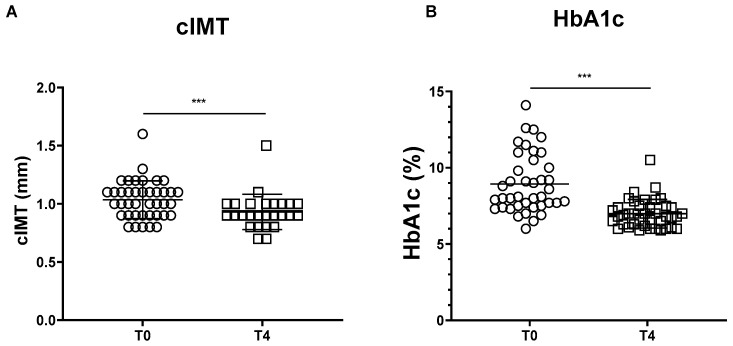
Effectiveness parameters. (**A**) Carotid intima-media thickness (mm) variation by visit, all patients (T0, *n* = 40; T4, *n* = 40). (**B**) HbA1c (%) variation by visit, all patients (T0, *n* = 40; T4, *n* = 40). cIMT: carotid intima-media thickness. Paired samples *t*-test; *** *p* < 0.001.

In the overall population, the cIMT showed a significant mean ± SD reduction of −0.14 ± 0.1, equivalent to −13.0 ± 9.2% reduction, compared with baseline after 4 months of semaglutide therapy (T0 mean value: 1.04 ± 0.16 mm, T4 mean value: 0.90 ± 0.14 mm; *p* < 0.001) (Figure 1A).

A significant reduction in the mean cIMT was also observed in the subgroup of patients with early diabetes (−10.4 ± 9.0%; T0 mean value: 0.97 ± 0.15 mm, T4 mean value: 0.86 ± 0.10 mm; delta cIMT: −0.11 ± 0.1; *p* = 0.003), medium diabetes (−13.5 ± 8.2%; T0 mean value: 1.07 ± 0.25 mm, T4 mean value: 0.92 ± 0.25 mm; delta cIMT: −0.14 ± 0.09; *p* < 0.001) and late diabetes (−14.3 ± 9.8%; T0 mean value: 1.06 ± 0.10 mm, T4 mean value: 0.91 ± 0.08 mm; delta cIMT: −0.16 ± 0.1; *p* < 0.001). There was no difference in the mean cIMT variation among the three subgroups (*p* = 0.417).

#### 3.2.2. HbA1c Levels

At the end of the observation period, the HbA1c mean levels showed a mean(±SD) reduction of −1.9 ± 1.4, equivalent to −19.9 ± 11.3% reduction, compared with the baseline after 4 months of semaglutide therapy (T0 mean value: 8.9 ± 1.9%, T4 mean value: 7.0 ± 0.9%; *p* < 0.001) (Figure 1B).

The same significant trend of HbA1c reduction was observed in patients with early diabetes (−21.2 ± 12.5%; T0 mean value: 8.8 ± 1.6%, T4 mean value: 6.8 ± 0.8%; delta cIMT: −2.0 ± 1.4; *p* < 0.001), in patients with medium diabetes (−19.2 ± 13.2%; T0 mean value: 9.2 ± 2.9%, T4 mean value: 7.1 ± 1.4%; delta cIMT: −2.0 ± 1.8; *p* = 0.009), and in patients with late diabetes (−19.4% ± 10.0%; T0 mean value: 8.9 ± 1.7%, T4 mean value: 7.1 ± 0.7%; delta cIMT: −1.9 ± 1.3; *p* < 0.001). There was no difference in HbA1c variation among the three subgroups (*p* = 0.95).

### 3.3. Secondary Outcomes

After 4 months of semaglutide therapy, a significant reduction in anthropometric parameters was observed in the overall population, including mean body weight (−5.6 ± 4.3 kg; *p* < 0.001), mean waist circumference (−3.6 ± 3.5 cm; *p* < 0.001), and mean BMI (−5.8 ±4.8 kg/m^2^; *p* < 0.001) (Table 2). Mean fasting glycemia decreased significantly compared to the baseline (−22.3 ± 26.7 mg/dL; *p* < 0.001), as did mean total cholesterol, triglycerides, and LDL cholesterol (−10.8 ± 16.1 mg/dL; *p* < 0.001; −4.3 ± 36.2 mg/dL; *p* = 0.021 and −11.1 ± 28.5 mg/dL; *p* < 0.001, respectively). HDL cholesterol slightly increased, although the difference did not reach statistical significance (*p* = 0.85; Table 2). Hepatic parameters also improved at the end of the observational period (alkaline phosphatase: −8.5 ± 22.2% U/I; *p* = 0.004; GGT, gamma-glutamyl transferase: −6.4 ± 21.5% UI/L; *p* = 0.011; ALT: −7.7 ± 22.4% UI/L; *p* = 0.002) (Table 2). Anthropometric, glycemic, and hepatic parameters and plasma lipids were homogeneous at the baseline in the three subgroups of patients with early, medium, and late diabetes except for the mean level of GGT, which was significantly higher in the early diabetes group (*p* = 0.031).

Subgroup analyses showed the same reduction trend in all anthropometric parameters (Supplementary Tables S2–S4). Fasting glycemia and total and LDL cholesterol significantly decreased in the early and late subgroups of patients, while in patients with medium-stage diabetes, a trend for reduction was observed (Supplementary Tables S2–S4). Regarding the hepatic parameters, a significant improvement in alkaline phosphatase and ALT was observed only in the late diabetes subgroup (*p* = 0.01 for both) (Supplementary Tables S2–S4). There was no difference in anthropometric, glycemic, and hepatic parameters and plasma lipid variation among the three subgroups (Supplementary Table S5).

A significant TG/HDL ratio reduction was also reported in the overall population after 4 months of treatment (−0.8 ± 2.0 mg/dL; *p* = 0.019).

### 3.4. Hepatic Steatosis and Fibrosis Markers and TyG Index

A statistically significant variation was found between the baseline and the 4-month visit for FLI, HSI, and TyG (Table 3).

The FLI was <30 in 1 (2.5%) patient at the baseline visit and in 4 (10%) patients at the 4-month visit (*p* = 0.250); a significant reduction was found in patients with an FLI > 60 at the 4-month visit (*n* = 22; 55%) compared with the baseline visit (*n* = 29; 72.5%; *p* = 0.039). The FLI was between 30 and 60 in 10 (25%) patients at the baseline visit and in 14 (35%) patients at the 4-month visit (*p* = 0.388). The number of patients with an HSI >36 was not statistically different at the baseline visit (*n* = 37; 92.5%) compared with the 4-month visit (*n* = 35; 87.5%; *p* = 0.50). The AST/ALT ratio was <1 in 33 (82.5%) patients at the baseline visit and in 26 (65.0%) patients at the 4-month visit (*p* = 0.065). The BARD score was <2 in 2 (5.0%) patients at the baseline visit and in 3 (7.5%) patients at the 4-month visit (*p* = 1.0). TyG was >4.55 in all patients at the baseline and 4-month visits.

#### Correlation Analyses

None of the considered scores/indices at the baseline correlate with cIMT variations. TyG score at the baseline was related to HbA1c levels at the 4-month visit (*p* = 0.049) (Supplementary Table S6). Variations in cIMT and HbA1c were unrelated to anthropometric, glycemic, and hepatic parameters and plasma lipids at the baseline (Supplementary Table S7).

### 3.5. Insulin Therapy during the Study Period

Five patients (12%) were on basal insulin therapy at the baseline. A total of 4 (80%) reduced insulin after the 4-month therapy with semaglutide. The mean ± SD baseline insulin dose was 22.4 ± 11.0 IU, and the mean ± SD insulin dose was 16.4 ± 10.6 IU at the end of the observational period. The mean dose reduction was equal to 6 ± 4 IU (*p* = 0.063).

## 4. Discussion

CV disease is the leading cause of morbidity and mortality in T2D patients [15,16]. Consequently, even if glycemic control is the primary outcome in managing T2D, a multifactorial approach to optimize CV risk factors is equally important [23,24].

Semaglutide reduced MACE in people with T2D at a high risk of CV disease in the SUSTAIN 6 trial, resulting in improved CV risk factors and increased life-years of the patients who practiced this therapy as an add-on to their practiced hypoglycemic therapy; this was probably due to the underlying CV risk of these patients and the time of initiation of treatment with semaglutide [10,16,25,26].

This 4-month retrospective, real-world study of T2D patients showed that injectable semaglutide significantly reduced HbA1c levels and cIMT, supporting the effect of this treatment on subclinical atherosclerosis as previously shown for some but not all GLP-1RAs [27,28,29,30]. This effect was independent of the duration of the disease, as a significant reduction in cIMT was observed in early-, medium- and late-stage diabetes.

We found a significant improvement in all the traditional CV risk factors in the overall population, including weight, BMI, waist circumference, and plasma lipids, as well as in the TG/HDL ratio, which is a marker of atherogenic small, dense (sd) LDL [31]. This latter finding was not previously reported and had a particular clinical significance since sdLDL is a key feature of diabetic dyslipidemia [32] and is closely associated with cardiovascular events [33]. We therefore highlight the clinical relevance of the reduction of atherogenic sdLDL found in the present study, which is in line with previous evidence found for liraglutide [34]; this mechanism may help to explain the beneficial cardiovascular outcome found with the use of injectable semaglutide [35]. The ongoing Semaglutide Anti-Atherosclerotic Mechanisms of Action Study (SAMAS) will further elucidate the role of sdLDL in type 2 diabetic patients receiving semaglutide in the oral formulation [36].

Semaglutide has been shown to be safe in adults and older patients with kidney or liver disorders requiring no dose modification [10]. In line with this observation, hepatic parameters also improved in our cohort. Moreover, we showed for the first time that the analysis of indices related to liver damage showed a positive effect of semaglutide on hepatic steatosis during the observational period (*p* < 0.001 for both FLI and HSI). Moreover, through correlation analyses, we found that hepatic fibrosis and steatosis indices and the anthropometric, hepatic, and glycemic parameters, as well as plasma lipids, were unrelated to the variations in cIMT and HbA1c. Otherwise, a positive correlation was observed between TyG and glycemia reduction (*p* = 0.049).

Our findings align with the literature evidence supporting the prominent place of semaglutide in treating T2D patients at high CV risk [12,26,37]. Indeed, data from various clinical trials highlighted the effectiveness of this treatment even compared with other GLP-1 agonists [14,15,38,39]. This can be explained by the higher homology of the new molecule to the GLP-1, which provides an effect more similar to native GLP-1 than other agonists with a lower degree of homology [40]. We cannot exclude that higher homology also plays a role in the cardiovascular outcome shown using the different GLP-1RAs [41].

Of note, our study is among the first to show the anti-atherosclerotic effects of injected semaglutide. Given that cIMT is a surrogate marker of early, subclinical atherosclerosis, our findings showing a regression of cIMT support the hypothesis, based on preclinical data, that the effect on atherosclerosis is one of the key CV protective mechanisms of semaglutide and propose a role for semaglutide in T2D patients with high CV risk [5,17,35]. Moreover, the significant reduction in the TG/HDL ratio further supports the CV protective activity of semaglutide [34,42]. For instance, in a recent preclinical study, semaglutide reduced the vascular uptake of tracers for activated macrophages and cellular metabolism imaging, supporting the hypothesis that the drug can reduce atherosclerotic inflammation through reduced activity of activated macrophages [43].

Overall, considering the observed semaglutide’s favorable effects on atherogenic lipoproteins and hepatic steatosis indices, our findings support the pleiotropic effect of semaglutide beyond glycemic control; they also underpin the European Heart Journal (EHJ) guidelines for diabetes and the 2022 update to the American Diabetes Association and the European Association for the Study of Diabetes consensus report, recommending GLP-1RAs as first-line therapy in patients with either high/very high CV risk or established CVD [1,44,45,46]. We can speculate that semaglutide could be considered a metabolic drug used for cardiometabolic prevention in T2D patients.

This study presents some limitations, such as the small sample size, the short duration of the observation phase, and the lack of a control group. However, even if most patients were treated with metformin, this therapy, as well as the use of ACEI/ARB in association with metformin, has been shown to have a negligible impact on cIMT and only a modest effect on anthropometric and lipid parameters [47,48,49]. For instance, a group of control patients treated with a hypoglycemic agent alone was not considered because in this case, being the only therapy practiced, it could be subject to dosage modifications and thus create a bias.

In conclusion, this study sheds light on the effects of injectable semaglutide, supporting the hypothesis that this treatment’s effect on atherosclerosis is one of the key CV protective mechanisms. Although further investigation is warranted to confirm our observations, this evidence could likely pave the way for more tailored and timely prevention and management of CV risk.

## Figures and Tables

**Table 1:** Baseline demographics and clinical characteristics.

Variables	Overall Population (*n* = 40)
Age (years)	66 ± 10
Male	26 (65)
Weight (kg)	88 ± 16
BMI (kg/m^2^)	32 ± 5
cIMT (mm)	1 ± 0.1
Disease duration (years):	14 ± 10
● Early diabetes	12 (30)
● Medium diabetes	9 (22)
● Late diabetes	19 (47)
Smoking status:	
● Yes	13 (32)
● Ex-smoker	16 (40)
● No	11 (28)
Familiarity with CV diseases	23 (57)
Comorbidities:	
● Hypertension	34 (85)
● Dyslipidemia	32 (80)
● Obesity	28 (70)
● Diabetes complications	11 (27)
Diabetes therapies:	
● Metformin	35 (87)
● Gliptins	3 (7)
● SGLT-2 inhibitors	7 (17)
● SU	9 (22)
● Pioglitazone	3 (7)
● Glargine	7 (17)
● Degludec	1 (2)
Concomitant therapies:	
● Β-blockers	18 (45)
● ACE inhibitors	13 (32)
● Calcium antagonists	11 (27)
● Diuretics	15 (37)
● AT1R blockers	20 (50)
Lipid-lowering drugs:	
● Statins	30 (75)
● Fibrates	2 (5)
● Omega-3	6 (15)
● Aspirin use	25 (62)

Data are presented as mean ± SD or *n* (%). ACE: angiotensin-converting inhibitor; cIMT: carotid intima-media thickness; CV: cardiovascular; SU: sulfonylurea.

**Table 2:** The effect of 4-month semaglutide therapy on the overall population (*n* = 40).

Variables	Baseline (Mean ± SD)	4 Months (Mean ± SD)	*p*-Value
Anthropometric parameters:			
Weight (kg)	88 ± 16	83 ± 14	<0.001
Waist (cm)	101 ± 11	97 ± 10	<0.001
BMI (kg/m^2^)	32 ± 5	30 ± 5	<0.001
Glycemic parameters:			
Glycemia (mg/dL)	176 ± 75	122 ± 22	<0.001
HbA1c (%)	9 ± 2	7 ± 1	<0.001
Plasma lipids:			
Total CHO (mg/dL)	179 ± 42	155 ± 29	<0.001
TG (mg/dL)	157 ± 90	131 ± 44	0.021
LDL CHO (mg/dL)	106 ± 40	88 ± 28	<0.001
HDL CHO (mg/dL)	42 ± 11	42 ± 9	0.058
Hepatic parameters:			
Alkaline phosphatase (U/L)	78 ± 45	66 ± 25	0.004
GGT (UI/L)	39 ± 24	34 ± 17	0.011
AST (UI/L)	22 ± 12	20 ± 7	0.067
ALT (UI/L)	29 ± 18	24 ± 11	0.002
Carotid IMT	1 ± 0.1	0.9 ± 0.1	<0.001

ALT: alanine aminotransferase; AST: aspartate aminotransferase; CHO: cholesterol; GGT: gamma-glutamyl transferase; HDL: high-density lipoprotein; IMT: carotid intima-media thickness; LDL: low-density lipoprotein; SD: standard deviation; TG: triglycerides.

**Table 3:** Steatosis markers, fibrosis markers, and triglyceride glucose index variation in the overall population (*n* = 40).

Variables	Baseline (Mean ± SD)	4 Months (Mean ± SD)	*p*-Value
Steatosis markers:			
● FLI	71.4 ± 21.3	61.6 ± 22.4	<0.001
● HSI	45.1 ± 6.0	42.3 ± 5.2	<0.001
Fibrosis markers:			
● AST/ALT ratio	0.83 ± 0.26	0.88 ± 0.19	0.173
● BARD score	2.8 ± 1.0	3.0 ± 1.0	0.115
● TyG index	6.4 ± 0.7	6.0 ± 0.4	<0.001

ALT: alanine aminotransferase; AST: aspartate aminotransferase; BARD: body mass index—AST/ALT ratio—T2D; FLI: fatty liver index; HSI: hepatic steatosis index; SD: standard deviation; TyG: triglyceride glucose.

## Data Availability

The datasets generated during and/or analyzed during the current study are available from the corresponding author upon reasonable request.

## Supplementary Materials

The following supporting information can be downloaded at: https://www.mdpi.com/article/10.3390/biomedicines11051362/s1, Table S1: Baseline demographics and clinical characteristics of patients stratified according to the disease duration; Table S2: The effect of 4-month semaglutide therapy on patients with early diabetes (*n* = 12); Table S3. The effect of 4-month semaglutide therapy on patients with medium diabetes (*n* = 9); Table S4. The effect of 4-month semaglutide therapy on patients with late diabetes (*n* = 19); Table S5: Anthropometric, glycemic, hepatic parameters and plasma lipids variation according to diabetes duration; Table S6: Correlation between hepatic steatosis markers (FLI, HIS), hepatic fibrosis markers (AST/ALT ratio, BARD score) and triglyceride glucose (TyG) index and changes in cIMT and HbA1c; Table S7: Correlation between anthropometric, glycemic, hepatic parameters and plasma lipids and changes in cIMT and HbA1c.
