# Supplementary material for: Effect of Semaglutide on Subclinical Atherosclerosis and Cardiometabolic Compensation: A Real-World Study in Patients with Type 2 Diabetes
Source: Biomedicines. 2023 May 4;11(5):1362. doi: 10.3390/biomedicines11051362 (PMC10216597; doi:10.3390/biomedicines11051362)
Supplement: Supplementary file 1 [file biomedicines-11-01362-s001.zip › biomedicines-2310173-supplementary.pdf]

Effect of semaglutide on subclinical atherosclerosis and cardiometabolic compensation: a real-world study in patients with type 2 diabetes

SUPPLEMENTARY MATERIAL

Supplementary Table S1: Baseline demographics and clinical characteristics of patients stratified according to the disease duration.

Variables	Early diabetes (n=12)	Medium diabetes (n=9)	Late diabetes (n=19)
Age (years)	61 ± 11	66 ± 10	69 ± 8
Male	10 (83)	4 (44)	12 (63)
Disease duration (years)	3 ± 2	11 ± 1	23 ± 7
Smoking status:			
• Yes	• 7 (58)	• 2 (22)	• 4 (21)
• Ex-smoker	• 2 (17)	• 5 (56)	• 9 (47)
Familiarity with CV diseases	8 (67)	5 (56)	10 (53)
Comorbidities:			
• Hypertension	• 10 (83)	• 9 (100)	• 15 (79)
• Dyslipidemia	• 8 (66)	• 9 (100)	• 15 (79)
• Obesity	• 9 (75)	• 6 (67)	• 13 (68)
• Diabetes complications	• 2 (16)	• 2 (22)	• 7 (37)
Concomitant therapies:			
• β-blockers	• 3 (25)	• 4 (44)	• 11 (58)
• ACE inhibitors	• 5 (41)	• 5 (56)	• 3 (16)
• Calcium antagonists	• 3 (25)	• 3 (33)	• 5 (26)
• Diuretics	• 4 (33)	• 2 (22)	• 9 (47)
• AT1R blockers	• 5 (41)	• 4 (44)	• 11 (58)
Lipid-lowering drugs:			
• Statins	• 6 (50)	• 9 (100)	• 15 (79)

• Fibrates<br>• Omega-3	• 1 (8)<br>• –	• –<br>• 3 (33)	• 1 (5)<br>• 3 (16)
Aspirin use	7 (58)	5 (56)	13 (68)

Data are presented as mean ± SD or n (%).

ACE: Angiotensin-converting enzyme; CV: Cardiovascular; SD: Standard deviation.

Supplementary Table S2: The effect of 4-month semaglutide therapy on patients with early diabetes (n=12).

Variables	Baseline (mean ± SD)	4 months (mean ± SD)	p-value
Anthropometric parameters			
Weight (kg)	90 ± 14	84 ± 10	0.006
Waist (cm)	102 ± 9	97 ± 7	0.001
BMI (kg/m^2)	31 ± 3	29 ± 3	0.003
Glycemic parameters			
Glycemia (mg/dL)	170 ± 50	118 ± 25	0.007
HbA1c (%)	9±2	7±1	<0.001
Plasma lipids			
Total CHO (mg/dL)	177 ± 37	158 ± 26	0.024
TG (mg/dL)	133 ± 40	129 ± 47	0.808
LDL CHO (mg/dL)	109 ± 33	88 ± 22	0.006
HDL CHO (mg/dL)	42 ± 8	44 ± 6	0.137
Hepatic parameters			
Alkaline phosphatase (U/l)	99 ± 66	77 ± 31	0.074
GGT (UI/L)	51 ± 32	41 ± 18	0.067
AST (UI/L)	24 ± 10	20 ± 6	0.058

ALT (UI/L)	30 ± 19	24 ± 11	0.054
Carotid IMT (mm)	1 ± 0.1	0.9 ± 0.1	0.003
SD: standard deviation; TG: triglycerides; CHO: cholesterol; AST: aspartate aminotransferase; ALT: alanine aminotransferase; GGT: gamma-glutamyl transferase.			

Supplementary Table S3. The effect of 4-month semaglutide therapy on patients with medium diabetes (n=9).

**Variables**	**Baseline (mean ± SD)**	**4 months (mean ± SD)**	**p-value**
Anthropometric parameters			
Weight (kg)	84 ± 17	81 ± 17	0.009
Waist (cm)	98 ± 11	94 ± 11	0.024
BMI (kg/m^2)	31 ± 8	30 ± 8	0.008
Glycemic parameters			
Glycemia (mg/dL)	177 ± 115	121 ± 16	0.133
HbA1c (%)	9±3	7±1	0.009
Plasma lipids			
Total CHO (mg/dL)	187 ± 60	152 ± 23	0.122
TG (mg/dL)	191 ± 122	131 ± 39	0.101
LDL CHO (mg/dL)	108 ± 54	82 ± 21	0.183
HDL CHO (mg/dL)	46 ± 14	46 ± 12	0.728
Hepatic parameters			
Alkaline phosphatase (U/l)	70 ± 42	59 ± 24	0.195
GGT (UI/L)	38 ± 20	34 ± 18	0.054
AST (UI/L)	25 ± 22	22 ± 12	0.418
ALT (UI/L)	30 ± 27	24 ± 15	0.190
Carotid IMT	1 ± 0.2	0.9 ± 0.2	<0.001
SD: standard deviation; TG: triglycerides; CHO: cholesterol; AST: aspartate aminotransferase; ALT: alanine aminotransferase; GGT: gamma-glutamyl transferase.			

**Supplementary Table S4. The effect of 4-month semaglutide therapy on patients with late diabetes (n=19).**			
**Variables**	**Baseline (mean ± SD)**	**4 months (mean ± SD)**	**p-value**
Anthropometric parameters			
Weight (kg)	89 ± 18	83 ± 15	<0.001
Waist (cm)	102 ± 12	98 ± 11	0.002
BMI (kg/m^2)	33 ± 4	30 ± 4	<0.001
Glycemic parameters			
Glycemia (mg/dL)	179 ± 68	124 ± 23	<0.001
HbA1c (%)	9±2	7±0.7	<0.001
Plasma lipids			
Total CHO (mg/dL)	176 ± 37	155 ± 34	<0.001
TG (mg/dL)	155 ± 94	131 ± 47	0.086
LDL CHO (mg/dL)	104 ± 40	89 ± 35	0.004
HDL CHO (mg/dL)	41 ± 11	39 ± 8	0.300
Hepatic parameters			
Alkaline phosphatase (U/l)	69 ± 22	63 ± 21	0.013
GGT (UI/L)	31 ± 17	29 ± 14	0.347
AST (UI/L)	19 ± 6	19 ± 5	0.777
ALT (UI/L)	27 ± 13	24 ± 10	0.015
Carotid IMT	1 ± 0.1	0.9 ± 0.08	<0.001
SD: standard deviation; TG: triglycerides; CHO: cholesterol; AST: aspartate aminotransferase; ALT: alanine aminotransferase; GGT: gamma-glutamyl transferase.			

Supplementary Table S5: Anthropometric, glycemic, hepatic parameters and plasma lipids variation according to diabetes duration				
**Variables**	**Delta**			**p-value**
	**Early diabetes (n=12)**	**Medium diabetes (n=9)**	**Late diabetes (n=19)**	
Anthropometric parameters				
Weight (kg)	-5.5 (5.5)	-3.5 (3.1)	-5.9 (5.0)	0.472
Waist (cm)	-4.7 (3.2)	-3.3 (3.6)	-3.3 (4.0)	0.603
BMI (kg/m^2)	-1.8 (1.6)	-1.2 (1.1)	-2.3 (2.1)	0.351
Glycemic parameters				
Glycemia (mg/dL)	-52 (54)	-56 (100)	-55 (57)	0.986
Plasma lipids				
Total CHO (mg/dL)	-19 (25)	-34 (59)	-21 (21)	0.548
TG (mg/dL)	-4 (53)	-60 (98)	-24 (58)	0.175
LDL CHO (mg/dL)	-21 (22)	-26 (50)	-14 (19)	0.600
HDL CHO (mg/dL)	+3.1 (6.7)	+0.7 (5.5)	-1.8 (7.5)	0.163
Hepatic parameters				
Alkaline phosphatase (U/l)	-22.0 (38)	-10.8 (22.8)	-6.5 (7.5)	0.243
GGT (UI/L)	-9.7 (16.5)	-4.4 (5.9)	-1.9 (8.6)	0.179
AST (UI/L)	-4.0 (6.6)	-3.2 (11.3)	-0.2 (3.2)	0.273
ALT (UI/L)	-5.4 (8.7)	-6.3 (13.3)	-3.1 (5.0)	0.594
SD: standard deviation; TG: triglycerides; CHO: cholesterol; AST: aspartate aminotransferase; ALT: alanine aminotransferase; GGT: gamma-glutamyl transferase.				

**Supplementary Table S6: Correlation between hepatic steatosis markers (FLI, HIS), hepatic fibrosis markers (AST/ALT ratio, BARD score) and triglyceride glucose (TyG) index and changes in cIMT and HbA1c**				
**Univariate analysis of variance**	**Dependent variable: cIMT**		**Dependent variable: HbA1c**	
	**F**	**p-value**	**F**	**p-value**
FLI	0.838	0.367	2.426	0.129
HSI	2.161	0.151	0.817	0.373
AST/ALT ratio	0.958	0.335	1.274	0.267
BARD score	0.162	0.690	0.052	0.821
TyG index	0.000	0.985	4.187	**0.049**
cIMT: carotid intima-media thickness; HbA1c: hemoglobin A1c; FLI: Fatty Liver Index; HSI: Hepatic Steatosis Index; TyG: trigyceride-glucose index. Bold values represent statistically significant p-value.				

**Supplementary Table S7: Correlation between anthropometric, glycemic, hepatic parameters and plasma lipids and changes in cIMT and HbA1c.**				
**Univariate analysis of variance**	**Dependent variable: cIMT**		**Dependent variable: HbA1c**	
	**F**	**p-value**	**F**	**p-value**
Total CHO	0.784	0.382	1.638	0.209
TG	0.927	0.324	2.053	0.161
LDL CHO	0.315	0.578	1.545	0.222
Alkaline phosphatase	0.085	0.73	0.821	0.371
GGT	0.937	0.340	0.571	0.455
AST	0.100	0.754	0.889	0.352
ALT	0.001	0.969	0.071	0.792
cIMT: carotid intima-media thickness; HbA1c: hemoglobin A1c; TG: triglycerides; CHO: cholesterol; GGT: gamma-glutamyl transferase; AST: aspartate aminotransferase; ALT: alanine aminotransferase;				
